# Evidence from Human and Animal Studies: Pathological Roles of CD8^+^ T Cells in Autoimmune Peripheral Neuropathies

**DOI:** 10.3389/fimmu.2015.00532

**Published:** 2015-10-15

**Authors:** Mu Yang, Corentin Peyret, Xiang Qun Shi, Nicolas Siron, Jeong Ho Jang, Sonia Wu, Sylvie Fournier, Ji Zhang

**Affiliations:** ^1^Department of Neurology and Neurosurgery, McGill University, Montreal, QC, Canada; ^2^The Alan Edwards Centre for Research on Pain, McGill University, Montreal, QC, Canada; ^3^Department of Microbiology and Immunology, McGill University, Montreal, QC, Canada; ^4^Faculty of Dentistry, McGill University, Montreal, QC, Canada

**Keywords:** Guillain-Barre syndrome, chronic inflammatory demyelinating polyneuropathy, CD8 T cells, macrophages, cytokines, co-stimulatory molecules, animal models

## Abstract

Autoimmune peripheral neuropathies such as Guillain-Barre Syndrome (GBS) and chronic inflammatory demyelinating polyneuropathy (CIDP) affect millions of people worldwide. Despite significant advances in understanding the pathology, the molecular and cellular mechanisms of immune-mediated neuropathies remain elusive. T lymphocytes definitely play an important role in disease pathogenesis and CD4^+^ T cells have been the main area of research for decades. This is partly due to the fact that the most frequent animal model to study autoimmune peripheral neuropathy is experimental allergic neuritis (EAN). As it is induced commonly by immunization with peripheral nerve proteins, EAN is driven mainly by CD4^+^ T cells. However, similarly to what has been reported for patients suffering from multiple sclerosis, a significant body of evidence indicates that CD8^+^ T cells may play a pathogenic role in GBS and CIDP disease development and/or progression. Here, we summarize clinical studies pertaining to the presence and potential role of CD8^+^ T cells in autoimmune peripheral neuropathies. We also discuss the findings from our most recent studies using a transgenic mouse line (L31 mice) in which the T cell co-stimulator molecule B7.2 (CD86) is constitutively expressed in antigen presenting cells of the nervous tissues. L31 mice spontaneously develop peripheral neuropathy, and CD8^+^ T cells are found accumulating in peripheral nerves of symptomatic animals. Interestingly, depletion of CD4^+^ T cells accelerates disease onset and increases disease prevalence. Finally, we point out some unanswered questions for future research to dissect the critical roles of CD8^+^ T cells in autoimmune peripheral neuropathies.

## Introduction

Autoimmune peripheral neuropathy, in the broadest sense, refers to a range of clinical syndromes mediated by aberrant immune response against self-antigens derived from peripheral nervous tissues including motor, sensory, and autonomic nerves. Guillain-Barré Syndrome (GBS) and chronic inflammatory demyelinating polyneuropathy (CIDP) are prototypical autoimmune peripheral neuropathies. The common incidence is about 1.6/100,000/year with a prevalence of 6–8.9/100,000 (1, 2). Clinically, GBS and CIDP patients present weakness, areflexia, and sensory deficits. Characterized by an acute and sudden onset, GBS is the most common cause of acute flaccid paralysis and represents a serious neurological emergency with 4% of GBS patients dying within the first year (3). CIDP is characterized by either chronic progressive, stepwise progressive, or relapsing weakness (1, 4). Although the majority of CIDP patients initially improve with immunosuppressive treatment, the relapse rate is about 50% (5). Over the last three decades, even with improved therapeutic options, both diseases still carry a severe prognosis as they are associated with significant mortality and sustained disability with 28% of patients requiring an assistive device to ambulate (6). The pathological changes in GBS and CIDP patients are characterized by inflammatory infiltration of both T cells and macrophages into peripheral nerves, as well as areas of demyelination, with or without axonal damage in peripheral nervous system (PNS) (4, 7).

Both humoral and cellular immune responses against antigen epitopes of Schwann cells, myelin and/or axons have been postulated to be responsible for autoimmune peripheral neuropathy. However, the extent and the details of the cascade leading to the peripheral nervous system damage are incompletely defined. Apart from the humoral immune response, T cells play a decisive role in the pathogenic sequence of immune-mediated nerve damage. Activated CD4^+^ T cells may operate by recruiting macrophages to exert damage on peripheral nerve tissue or may help B cells to produce antibodies against peripheral nerve components, thereby inducing complement activation. In addition to mediating local inflammatory response as CD4^+^ T cells do, activated CD8^+^ T cells, acting as cytotoxic effector cells, can contribute directly to the damage of both myelin and axons.

From recent research, CD8^+^ T cells emerged as important players in multiple sclerosis (MS) pathogenesis. Increasing evidence indicates that CD8^+^ T cells predominate and outnumber CD4^+^ T cells in all MS lesions, regardless of disease stages (8). Furthermore, depletion of CD4^+^ T cells did not show any therapeutic effect in MS patients (9), but when all T cells were targeted, a significant reduction in MS relapse was observed (10). In fact, infiltration of nervous tissues by CD8^+^ T cells has been observed in various classical neurodegenerative disorders, such Amyotrophic Lateral Sclerosis, Alzheimer disease, and Parkinson’s disease (11–13). However, due to the rare incidence of the diseases and the shortage of appropriate animal models, our understanding on the relative contribution of CD4^+^ and CD8^+^ T cells in the pathogenesis of GBS and CIDP is very much limited. Because experimental allergic neuritis (EAN), the most used animal model for the study of autoimmune peripheral neuropathy, is driven mainly by CD4^+^ T cells (14, 15), CD4^+^ T cells have been the main area of research for decades. In this perspective article, we will summarize clinical studies pertaining to the presence and potential role of CD8^+^ T cells in autoimmune peripheral neuropathy. We will also discuss the findings from various current available animal models. Emphasis will be given to our most recent studies using B7.2 transgenic (L31) mice where animals develop spontaneous autoimmune peripheral neuropathy and CD8^+^ T cells are the major players (16). L31 mice provide a unique opportunity to investigate underlying mechanisms of CD8^+^ T cell-mediated autoimmune peripheral neuropathy.

## Putative Relevance of CD8^+^ T Cells in Autoimmune Peripheral Neuropathy: Evidence from GBS and CIDP Patients

In humans, two-thirds of GBS are preceded by infections with viruses or bacteria especially *cytomegalovirus* (CMV) and *Campylobacter jejuni* (17, 18). Although not as frequent as in GBS, the onset and the relapse of CIDP can also be triggered by infections or immunization (5, 19). Biochemical and histopathological evidence suggests the potential involvement of T cells in the pathogenesis of these autoimmune peripheral neuropathies. The levels of soluble interleukin-2 receptors (20) and the frequencies of activated T cells were elevated in the serum of GBS (21) and CIDP (22) patients. Multifocal infiltration of lymphocytes were also found in post mortem and biopsy specimens of most GBS and CIDP cases (23). However, the specific targets and actors (CD4^+^ and/or CD8^+^ T cells) of the immune response remain uncertain. Although some discrepancies exist, several data imply putative relevance of CD8^+^ T cells in the pathogenesis of autoimmune peripheral neuropathies. For instance, the mean proportion of CD8^+^ T cells significanly increased in the blood GBS patients compared to the control group of healthy donors (24); CD8^+^ T cells were found to out-number CD4^+^ T cells at the lesion sites of CIDP (25) and GBS (26) patients. Interestingly, Sindern et al. (27) revealed that the composition of the T cell subpopulations in the blood of GBS patients depends in particular on the nature of the proceeding infection. They found that in GBS patients with evidence of recent CMV infection, the proportion of CD8^+^ T cells were abnormally high whereas the proportion of CD4^+^ T cells were abnormally low; in contrast, CD8^+^ T cells were abnormally low in GBS patients with evidence of *C. jejuni* infection. Furthermore, they reported an increase of activated cytotoxic/suppressor T cells (CD8^+^CD38^+^) in progressive and plateau phases of GBS, which was normalized in the recovery phase. More direct evidence in supporting pathogenic contribution of CD8^+^ T cells in CIDP was provided by two recent studies. Mausberg et al. (28) reported that CD8^+^ T cells exhibited a much broader clonal activation pattern than CD4^+^ T cells in the blood of CIDP patients. In addition, IVIg treatment, which was beneficial to patients, normalized the distorted CD8^+^ T cell repertoire and reduced the number of highly activated Vβ elements within the CD8^+^ T cell population. Another study by Schneider-Hohendorf et al. (29) reported that T cells in CIDP biopsies showed strong monoclonal and oligoclonal restrictions in their T cell repertoire, which were reflected in the patients’ blood CD8^+^ T cell pool. Taken together, these data support the hypothesis of an antigen driven, CD8^+^ T cell-mediated attack against nerve tissues, even if the target (antigen) of this immune response still remains to be identified.

## CD4^+^ and CD8^+^ T Cells in Autoimmune Peripheral Neuropathy: Insights from Animal Models

### CD4^+^ T Cells in EAN and NOD B7.2KO Mice

First described in 1955, EAN can be induced either by immunization with myelin peptide or by active transfer of antigen sensitized T cells in rats, mice, rabbits, and guinea pigs (14, 15). Many of our current knowledge of immune-mediated mechanisms of demyelination were primarily based on studying EAN, the animal model for human GBS and CIDP (30). EAN resembles many of the clinical and electrophysiological aspects of human GBS/CIDP. The pathological hallmark of EAN consists of infiltration of peripheral nerves by lymphocytes, predominantly CD4^+^ T cells, and macrophages with segmental demyelination and some axonal damage. Previous studies have shown that EAN belongs to the group of CD4^+^ T cell-mediated autoimmune diseases that can be transferred to naïve animals by CD4^+^ P2-reactive T cells (31). While EAN has provided valuable information regarding immunopathogenic mechanisms, it has been criticized for its artificial manipulation resulting in the bias towards CD4^+^ T cells. Development of spontaneous autoimmune peripheral neuropathy in B7.2 deficient NOD mice (32) introduced another tool for mechanistic studies. The individual role of CD4^+^ T cells vs. CD8^+^ T cells in the pathogenesis of the disease has been carefully investigated. While transfer of purified CD4^+^ T cells isolated from affected animals induced the disease in NOD⋅SCID mice, the transfer using preparation of CD4^+^ T-depleted cells failed in triggering the disease (32). On the other hand, spontaneous autoimmune neuropathy was rapidly induced in NOD⋅SCID mice after transfer of CD8^+^-depleted preparation from affected mice (32). These results highlight the necessary and sufficient role of CD4^+^ T cells in the effector phase of this autoimmune disease model.

### CD8^+^ T Cells in B7.2 Transgenic L31 Mice

We recently established a novel and clinically relevant animal model of spontaneous autoimmune peripheral polyneuropathy in which CD8^+^ T cells play a critical role (16). Transgenic mice with constitutive expression of the co-stimulator B7.2 were originally generated by placing the murine B7.2 cDNA under the transcriptional control of a MHC-I promoter and an immunoglobulin enhancer (33). Among multiple transgenic lines, Line 31 (L31) mice spontaneously developed neurological symptoms at the age of 4–6 months (34). Massive infiltration of CD8^+^ T cells and B7.2 high expression macrophages were found in inflamed nerves (16). Deficiency in CD4^+^ T cell generation accelerated disease onset and increased disease prevalence (35).

**Figure 1:**
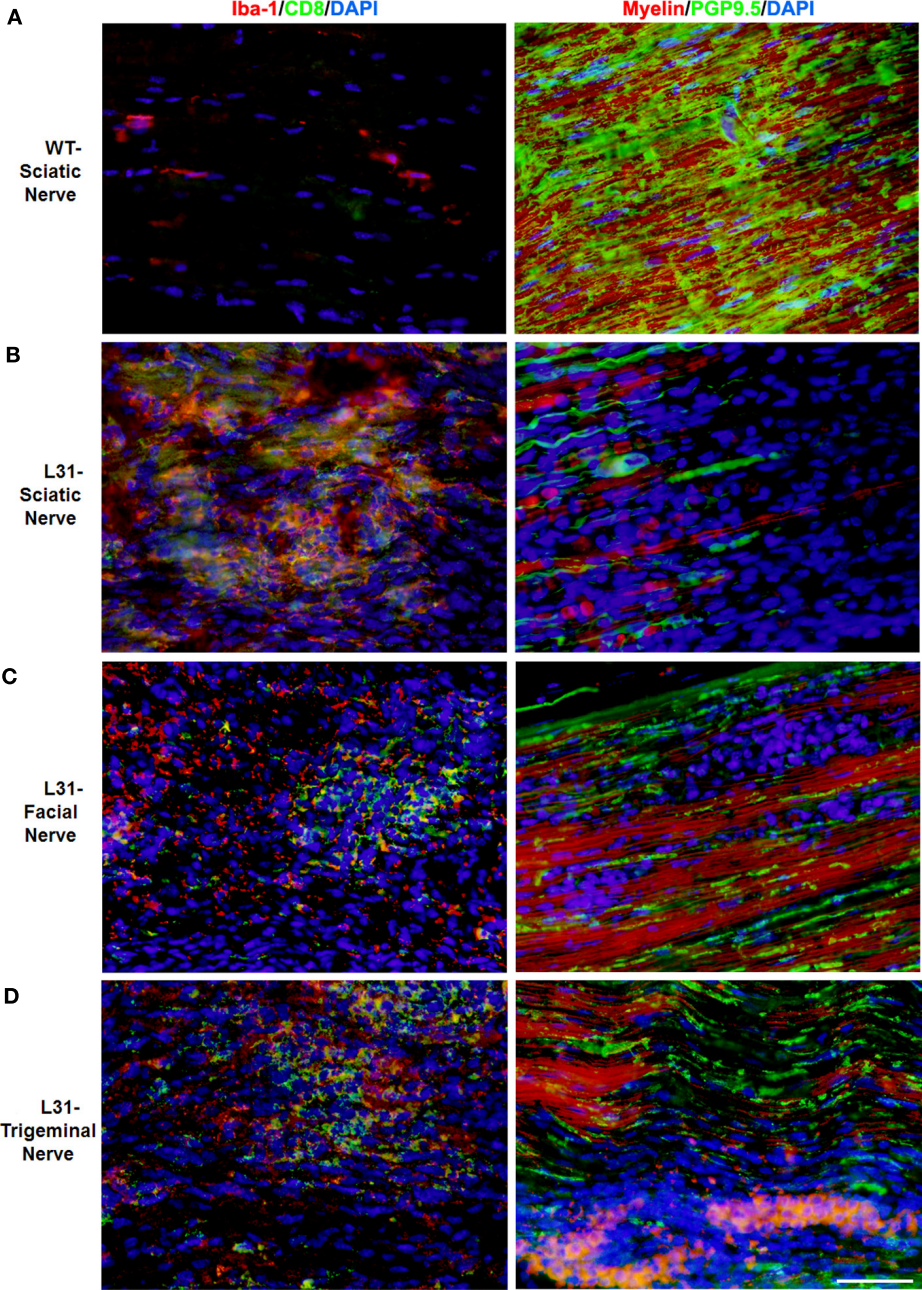
**Pathological changes in peripheral nerves of L31 mice after disease onset**. Immune cell infiltration was revealed by immunohistochemistry analysis using antibodies against CD8 and Iba-1 (macrophages), while demyelination and axonal damage were detected with antibodies against PGP9.5 and fluoromyelin, respectively. **(A)** Only few resident macrophages and no CD8^+^ T cells were detected in sciatic nerves of wild type (WT); there were massive infiltration of Iba-1^+^ macrophages and CD8^+^ T cells as well as severe demyelination and axonal damage in sciatic **(B)**, facial **(C)**, and trigeminal nerves **(D)** of symptomatic L31 mice (L31). Scale bar: 50 μm.

L31 mice and L31mice deficient in CD4^+^ T cells (L31/CD4KO) exhibit motor and sensory deficits, including weakness and paresis of limbs, numbness to mechanical stimuli, and hypersensitivity to thermal stimulation. Stereotypic pathological changes, including demyelination, axonal damage, and infiltration of CD8^+^ T cells and macrophages were found not only in sciatic nerve of symptomatic L31 mice (Figures 1A,B) but also in cranial nerves, e.g., facial (Figure 1C) and trigeminal (Figure 1D) nerves. However, it is worth noting that only limited inflammatory reaction and demyelination were observed in the spinal cords of diseased transgenic mice (16), which is consistent with reports from GBS patients (36). In addition, there was no tissue destruction or immune cell infiltration in other organs (34). The mechanism by which the autoimmune cascade is initiated in L31 mice remains elusive. We hypothesize that PNS selectivity could be determined, in one hand, by the distribution of B7.2 expression. In this model, B7.2 expressed constitutively only on resident microglia and macrophages of the nervous system, but not on APCs of any other tissues. We have shown that this B7.2 expression on the nervous tissues is an absolute requirement for susceptibility to disease development (34). On the other hand, as we detected immune infiltrates in pre-symptomatic animals in the DRG and spinal roots where the blood nerve barrier is fenestrated under physiological conditions (16), it implies that the virtual absence of the barrier in these peripheral nerve structures allows CD8^+^ T cells, during immune patrolling, to encounter resident macrophages overexpressing B7.2. The CD8^+^ T cells are reactivated by PNS self-antigens presented by resident macrophages. They are responsible to initiate PNS antigen specific autoimmune response. Although molecular targets for the CD8^+^ T cells are not fully defined, MHC class I is expressed constitutively on rodent Schwann’s cells and they are able to activate CD8 T cells (37). MHC class I upregulation has been observed on Schwann’s cells in sural nerves of GBS patients (26, 38). We are currently assessing MHC I expression by the cellular elements of the peripheral nerves of L31 mice. The demonstration of direct contact between T cells and MHC class 1–expressing target cells (neurons and/or Schwann’s cells) would be a strong evidence for a CD8^+^ T cell–mediated attack.

**Figure 2:**
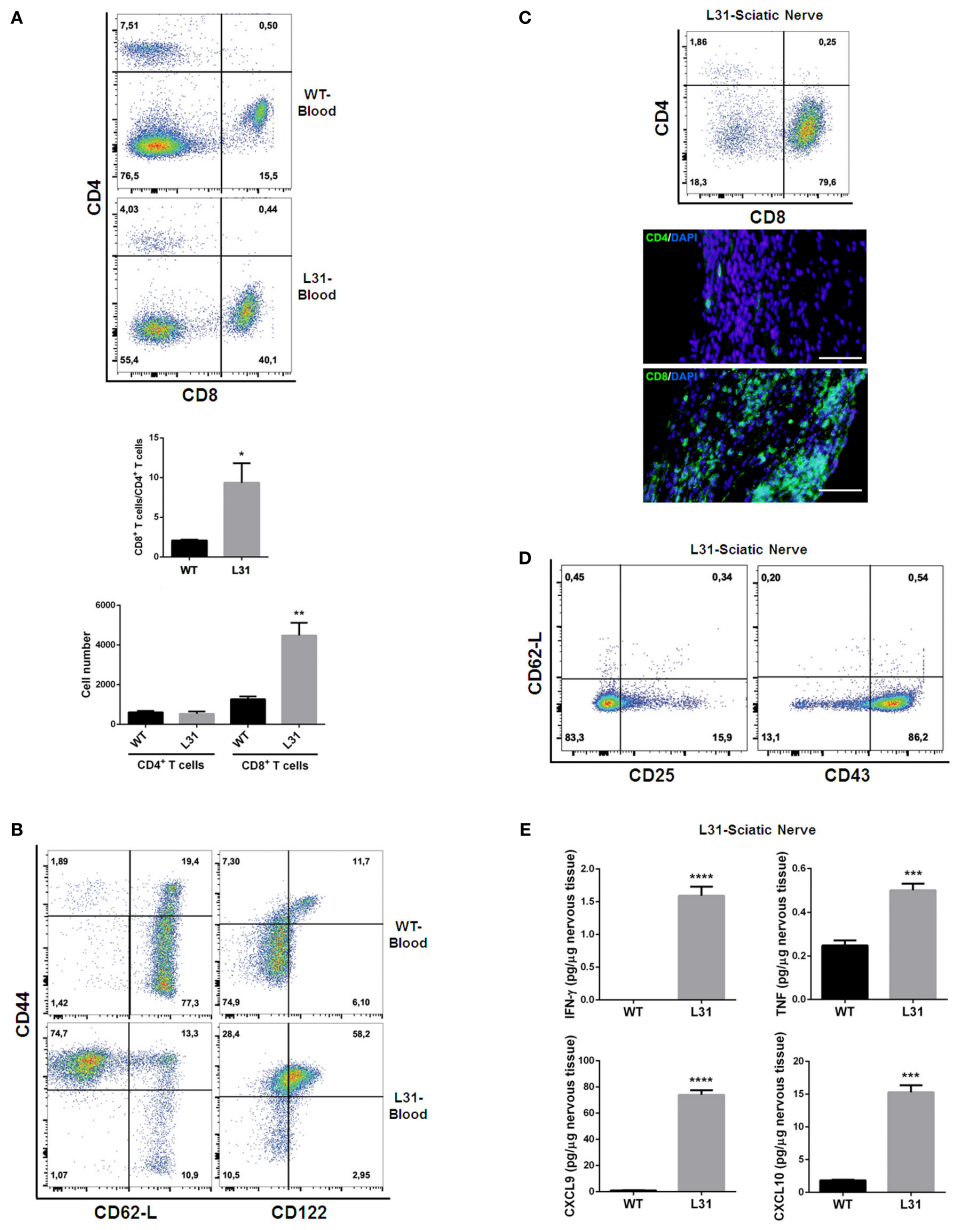
**T cell phenotypes in symptomatic L31 mice**. Flow cytometry analysis revealed that the ratio of CD8^+^ T cells vs. CD4^+^ T cells was significantly higher in the circulating system of symptomatic L31 mice (L31) by comparing with that in wild type (WT) mice, this enhancement was derived from the dramatically increase of CD8^+^ T cell absolute numbers in the blood of L31 mice **(A)**. CD8^+^ T cells from the blood of symptomatic L31 mice exhibited memory/effector phenotypes, which were determined by CD44^hi^CD62-L^lo^CD8^+^, as well as the expression levels of CD122 in CD44^hi^CD8^+^ population **(B)**. In parallel, both flow cytometry and immunohistochemistry analyses demonstrated that infiltration of T cells in affected sciatic nerves was predominated by CD8^+^ T cells **(C)**, Scale bar: 50 μm. The majority of infiltrated CD8^+^ T cells have low expression of CD62-L and high expression of CD43, but without the high levels of CD25 expression, which is a typical phenotype of activated T cells **(D)**. Cytokine/chemokine expression was analyzed using LUMINEX assay. The results indicated a significantly increase of both pro-inflammatory (IFNγ and TNF) and chemokines (CXCL9 and CXCL10) in affected sciatic nerves of symptomatic L31 mice **(E)**. *n* = 3–4/group, ***p < 0.001; ***p < 0.0001.

In the blood of diseased L31 mice, the ratio of CD8^+^/CD4^+^ T cells was about five times higher than that in wild type (WT) mice, which is mainly due to the significant increase of absolute CD8^+^ T cell numbers in the circulation (Figure 2A). Furthermore, these CD8^+^ T cells exhibited activated phenotypes, as the majority of these CD8^+^ T cells expressed high levels of CD44. A greater part circulating CD8^+^CD44^hi^ T cells exhibited CD62-L^lo^ phenotype (Figure 2B) that is different from the small amount of central memory CD8^+^ T cells (CD44^hi^CD62-L^hi^) in the blood of WT littermates, suggesting that CD8^+^ T cells in symptomatic L31 mice had already encountered antigens. The increase of CD8^+/^CD44^hi^/CD122^hi^ population in the blood (Figure 2B) entailed a group of effecter CD8^+^ T cells, while the increase of CD8^+^/CD44^hi^/CD122^lo^ cell population (Figure 2B) suggested the existence of atypical memory CD8^+^ T cells such as those found during chronic viral infections which also exhibit low levels of CD122 expression (39).

In affected sciatic nerves of L31 mice, T cell infiltration was skewed strikingly towards CD8^+^ T cells (Figure 2C). These infiltrated CD8^+^ T cells displayed activated phenotypes characterized by CD62-L^lo^/CD43^hi^ expression, but did not exhibit high levels of CD25, which is usually considered as an early T cell activation marker (Figure 2D). Furthermore, the data from the diseased nerves of L31/CD4KO revealed a significant increase of cytokines (IFNγ, TNF) and chemokines (CXCL9, CXCL10) (Figure 2E). These cytokines/chemokines released by CD8^+^ T cells and/or macrophages are essential in promoting recruitment and interactions between different subsets of immune cells. The fact that L31 mice deficient in IFNγ receptors were completely resistant to disease development (35) supports the requirement of IFN-γ, mainly derived from CD8^+^ T cells in disease pathogenesis.

Macrophages are also abundantly accumulating in peripheral nerves of symptomatic L31 mice. It remains to determine whether and to what extent these cells are required for disease pathogenesis. We are particularly interested in assessing the critical role of macrophage phagocytosis in Ag presentation, disease initiation and macrophage-associated oxidative burst-mediated damage of the nervous tissue. Other mechanisms involving macrophages, such as antibody-dependent cell-mediated-cytotoxicity could also be of relevance.

To summarize, the massive infiltration of T cells in diseased nervous tissues of L31 mice is CD8^+^, not CD4^+^ T cells. CD8^+^ T cells in the blood and in the nerves of diseased L31 display memory/effector phenotypes. Lack of T cells prevents disease development in L31 mice (34). L31 mice deficient in CD4^+^ T cells have an accelerated disease onset and an increased disease penetrance (35). Interestingly, L31 mice have an increased number of Foxp3-expressing CD4^+^ T cells in their lymphoid organs, which display an activated phenotype (unpublished data). Initiation of disease requires Ag specific CD8^+^ T cell activation since L31 mice with an almost unique TCR specificity expressed by CD8^+^ T cells (L31/OT-1 mice) never developed disease (35). Hence, CD8^+^ T cells arise as key players in disease pathogenesis in L31 B7.2 transgenic mice. Different from EAN where CD4^+^ T cells are the main culprits in mediating neuropathy, L31 mice provide a unique opportunity to investigate the involvement of CD8^+^ T cells in autoimmune peripheral neuropathy. In L31 mice, CD4^+^ T cells may function as regulators in the disease pathogenesis.

## Lessons from Animal Models for Human Autoimmune Peripheral Neuropathy

Whether induced or spontaneous, no single animal model perfectly mimics a human disease. These models represent only particular aspects of a complex human autoimmune disease, and not all animal response patterns are replicated in the human immune system. Among the most frequently used animal models of autoimmune peripheral neuropathy that we discussed above, EAN and NOD B7.2KO mice have provided valuable information in the principles of CD4^+^ T cell-mediated autoimmunity and in the development of clinically applicable immunotherapies. However, caution should be taken in the interpretation of results, especially, EAN, which is induced using complete Freund’s adjuvant. L31 mice offer a unique and excellent tool to investigate CD8^+^ T cell-mediated spontaneous autoimmmune neuropathy. As epidemiological studies have provided strong evidence on the involvement of viral infection in the onset of GBS and CIDP, this model might be essential in elucidating their viral etiology. However, L31 mice do not recover without intervention, suggesting this model is not appropriate for examining the mechanism of self-recovery in GBS patients. On the whole, autoimmune peripheral neuropathy is neither a single disease nor a disease stemming from one etiology; instead these are syndromes with multiple variants and very complex mechanisms. Thus, while recognizing the limitations of extrapolating findings from each animal model to human disease, what is important is the integration of the diverse data generated from different models into coherent framework for understanding the entirety of autoimmune peripheral neuropathy.

## Future Directions: Questions Need to be Addressed

Although the potential contribution of CD8^+^ T cells in autoimmune neuropathy, including MS, GBS, and CIDP, has brought considerable attention in recent years, our knowledge on CD8^+^ T cells is still very much limited. Why do so many people have viral or bacterial infections, but only a small population develop GBS/CIDP? Where is the site of the abnormality that initiates the disease process? What are the key molecules or cells that determine the fate of the disease? Why do some patients recover spontaneously, while others do not? Whether and to what extent CD8^+^ T cells are engaged in different stages of the diseases, and whether CD8^+^ T cells can be targeted for disease prevention and effective treatment? Use of appropriate animal models should help answer these questions and decipher the role of CD8^+^ T cells in human autoimmune peripheral neuropathy.

## Statement on Animal Ethics

All experiments were in accordance with the guidelines of the Canadian Council on Animal Care, and approved by the animal care committee of McGill University (Permit #5533).

## Conflict of Interest Statement

The authors declare that the research was conducted in the absence of any commercial or financial relationships that could be construed as a potential conflict of interest.
